# Construction of a patient decision aid for the treatment of uncomplicated urinary tract infection in primary care

**DOI:** 10.1186/s12875-021-01374-3

**Published:** 2021-01-26

**Authors:** Yves-Marie Vincent, Adèle Frachon, Clotilde Buffeteau, Guillaume Conort

**Affiliations:** Département de Médecine Générale, Université de Bordeaux, Collège Sciences de la santé, 146 rue Léo Saignat, 33076 Bordeaux Cedex, France

**Keywords:** Uncomplicated urinary tract infection, Cystitis, Shared decision-making, Patient decision aid, Patient-centred care, Primary care, Antibiotic, Focus group

## Abstract

**Background:**

Uncomplicated urinary tract infection (uUTI) is very common among women in primary care. The risk of developing pyelonephritis remains low after uUTI, nonetheless, empiric antibiotic therapy is frequently prescribed for symptomatic purposes. This may lead to adverse effects and antibiotic resistance. Furthermore, patients may express the will to limit the use of antibiotics. Some European countries recommend discussing a delayed prescription with the patient and developing a shared decision. The aim of this study is to create a patient decision aid (PtDA) used in primary care settings to make a shared decision between practitioners and women about whether or not to treat uUTI with antibiotics.

**Methods:**

We followed the steps recommended by the International Patient Decision Aids Standards, with a scoping phase, a design phase (including focus groups and literature review), and an alpha-testing phase. A steering group, made of patients and physicians, met throughout the study to develop a prototype PtDA.

**Results:**

The information included in the PtDA is the definition of uUTI, information on the options, their benefits, risks, and consequences, based on a review of the literature. The results of the focus group made possible to determine the patient’s values and preferences to consider in decision-making, including: the discomfort felt, the impact on daily life, patients’ perceptions of antibiotics, and the position relative to the risk of adverse effect. The choices in presentation, organisation and design are the result of the work of the steering group, improved by feedback from alpha testing. We confirmed the need for shared decision-making and the equipoise in this situation.

**Conclusions:**

We developed a PtDA to be used in primary care for sharing decision on the use of antibiotic in uUTI. It needs to be validated in a beta-testing phase, with complementary advice from peers, and then tested in a clinical study comparing its use with the systematic prescription approach.

## Background

Uncomplicated urinary tract infection (uUTI), or cystitis, is among the leading reasons for infectious disease consultation in primary care [1]. Nearly half of women report at least one episode of uUTI during their lifetime [2].

There is no standard definition of uUTI internationally accepted. In France, uUTI in women is defined as an acute urinary tract infection in adult under 75 years-old, without signs of pyelonephritis or risk factor for more severe disease (pregnancy, urogenital abnormalities, immunodeficiency, frailty).

Acute cystitis affects several facets of women’s life: social and intimate relationships, self-esteem, and ability to work. Depending on the situation, the impact can vary from a woman to another: from small discomfort to severe disability impacting personal and/or professional life [3, 4]. Nonetheless, cystitis can resolve spontaneously [5] with a rare risk of pyelonephritis [6]. In this context, some patients prefer either to discuss the advantages and disadvantages of antibiotics with their physicians [7] or even to avoid antibiotic uUTIs treatment [8]. This lead several authors to suggest a shared decision-making approach in uUTI [9, 10].

Currently, the systematic and immediate prescription of empiric antibiotic treatment is often recommended to reduce symptoms [11], even though physicians tend to overestimate patients’ desire to take antibiotics [8].

First line antibiotic treatment often differs from one country to another [12]. In France, the French-Language Infectious Pathology Society (*Société de Pathologie Infectieuse de Langue Française)* recommends fosfomycin [13], but it is important to note that, frequently, the antibiotic prescribed empirically does not match pathogen sensitivity on the microbiological results [14]. The effectiveness of a systematic antibiotic strategy on the infection must be put into perspective with potential adverse effects like mycosis, headache, dizziness and digestive disorders, and with the risk of resistance emergence [15]. In France, fluoroquinolones resistant *E. coli* are found in 3 to 7% of UTI in primary care [16, 17]. The resistance rate of bacteria responsible for uUTI is higher during the year following the prescription of an antibiotic for urinary tract infection in the primary care setting [18, 19]. Additionally, research indicates that compliance with prescribed treatment is variable [4].

Few countries already recommend other strategies such as delayed treatment. Thus, in the United Kingdom and the Netherlands, guidelines for uUTI suggest that physicians discuss the options with the patient to delay antibiotic treatment [20, 21].

Patient decision aids (PtDAs) are defined by the International Patient Decision Aids Standards (IPDAS) as “*tools designed to help people participate in decision making about health care options”,* providing *“information on the options”* and helping patients *“clarify and communicate the personal value they associate with different features of the options*” [22]. They make the process easier to inform patients, explore different treatment options [23] and have a positive effect on the diffusion of information, clarifying the patients’ values, risk perception, and involvement in the decision-making process [24]. These PtDAs are particularly relevant when linked to clinical recommendations [25].

A Dutch study reported that general practitioners who used shared decision-making with their patients prescribed fewer antibiotics in women under the age of 40 consulting for UTI [26]. In accordance with its guidelines, the National Institute for Health and Care Excellence (NICE) published a decision aid to help health care professionals to explain and discuss antibiotic treatment options to patients with uUTI, [27]. However, this decision aid does not incorporate all the dimensions recommended by the IPDAS [22], such as help to clarify values, visual presentation of numeric probabilities or guidance in deliberation and in communication. In addition, to date, there is no PtDA available for the treatment of uUTI for a French-speaking person.

The objective of this study is to develop a patient decision aid for shared decision-making for the treatment of uUTI in primary care setting.

This PtDA is part of a research project that aims to compare antibiotic consumption 14 days post-randomization between current practice and the use of a PtDA in the management of uUTI.

## Methods

IPDAS reviewed the development process of PtDAs to provide some empirical pragmatic guidelines [28]. They recommend documenting each step in an iterative process involving both physicians and patients, and to give a checklist to help future PtDAs developments.

**Fig. 1:**
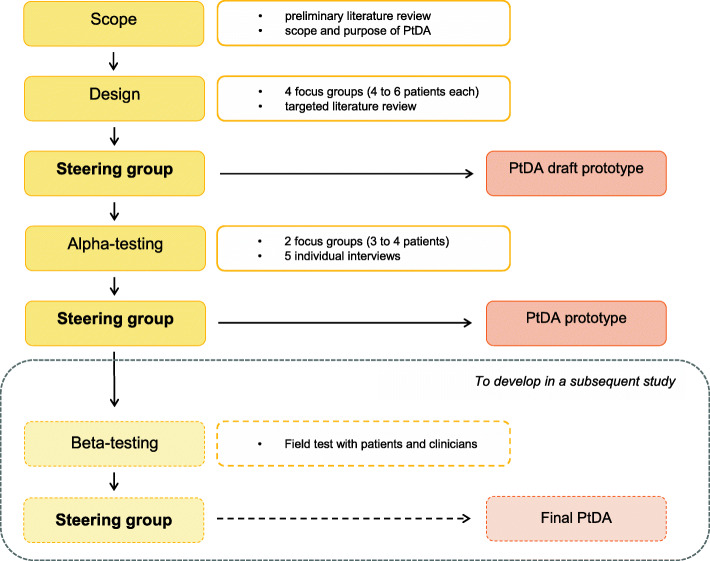
Development process, adapted from IPDAS

The development of our PtDA was conducted according to these recommendations (Fig. 1). This study focused on the first four stages of development: scoping, steering group, design and alpha testing, in order to acquire enough information to launch the beta-testing phase. This last step of development has to be carried out in clinical practice and therefore requires another recruitment and study.

### Initial design

#### Literature review

Two researchers (GC, YMV) conducted a preliminary literature review on antibiotic prescription in uUTI in primary care settings, using MEDLINE (see annex) for search strategy) and in the Cochrane databases. The results were screened using the title and abstract, followed by full text assessment. Selection criteria were randomised controlled trials comparing an antibiotic treatment with either another antibiotic, a placebo, or symptomatic treatment in the primary care settings or meta-analysis including primary care settings, the description of efficacy, side effects and risks with or without antibiotic treatment. This review aimed to define the decision targeted in the PtDA, its relevance and the risks for patients. Further relevant studies were included after inspecting the reference lists in selected studies. This literature review was then updated by a third researcher (AF), targeting the outcomes identified by the steering group after the design phase and after the alpha-testing phase. These outcomes were the natural history of uUTI, the duration of symptoms with antibiotics, the complication rate, the side effects rate, the recurrence and resistance rate, the alternative treatment options and the patient’s acceptance of health care. These elements were summarised in a table and presented to the steering group. The type of the study, date of publication, the type of population with inclusion criteria, and the type of treatment were also summarised, and relevant figures were added.

We finally searched for examples of PtDAs on the internet with inventories of such tools, like the Ottawa hospital [29] and the Université Laval [30]. We completed this work with clinical practice guidelines in countries with care settings similar to France, to determine the first line treatment and the association of guidelines with PtDAs.

#### Focus groups

We used a qualitative method with focus groups [31]. The objective was to explore and clarify patients’ expectations regarding the pieces of information and the format and design of the patient decision aid in the context of uUTI.

##### Participants

The inclusion criteria were adult women, with or without a history of uUTI. We used a purposive sampling approach, in order to maximise variation on socio-demographic criteria. The criteria for variation were the participants’ place of residence, socio-professional status, and age. The initial recruitment was carried out in patient support and community groups. This did not result in a sufficient number of patients to form focus groups. Additional recruitment was then conducted in rural and urban private practices, during consultations, or with posters. Four focus groups were created, each including between four and six women.

##### Interview guide

A semi-structured interview guide was developed by the four investigators, based on the SUNDAE checklist [32], exploring the following elements:

- knowledge and experience of uUTI
- the physical, emotional, and social impact of uUTI
- knowledge and expectations regarding different treatment options
- expectations on the format, terms, and presentation of the PtDA

Open questions, created based on the literature review, were asked to encourage the emergence of idea.

##### Implementation of focus groups

The focus groups took place in general practices outside the health care setting or on the premises of patient support and community groups. They were audio-recorded. Prior to the focus group, the participants were asked to fill out a short questionnaire to obtain socio-demographic data, including their age and professional status. Each focus group began with a brief presentation to the participants of the concept of shared decision-making and of the purpose of the study. Exchanges during focus groups were facilitated by a moderator experienced in conducting such interviews (GC and YMV, then AF and CB). The moderator was seconded by a researcher in charge of noting non-verbal communication and ensuring that no theme or participant was overlooked by the moderator.

##### Inductive analysis

The focus groups were entirely transcribed and anonymised. The data analysis was carried out with the R software (version 3.5.1, RQDA package). We followed an inductive content analysis approach, using grounded theory as described by Kitzinger et al. [31, 33]. After a global reading of the verbatims, the minimum meaning units were identified. They were then coded according to the different aspects relevant to patient decision-making and were labelled using shortened titles. Double coding was carried out independently by two researchers (AF and CB). Doubts or disagreements were discussed before pooling the final analysis. These meaning units were grouped into categories using thematic analysis, and then presented and discussed during steering meetings in order to establish the specifications of the PtDA.

##### Ethics

Before each focus group, participants signed an informed consent agreement. They were informed of the objective of the research project. The anonymity and confidentiality of the data were ensured by anonymizing the recordings during transcription and then by deleting the recordings at the end of the study. The project received a favorable opinion from the National Commission for Data Protection (*Commission nationale de l’informatique et des libertés*) on 13 March 2018.

### Draft version of the prototype

The steering group consisted of four physicians (the researchers) and three patients. The patients were recruited through patient support and community groups and general practices. Their written consent and socio-demographic characteristics were collected.

The group used the checklist established by IPDAS [22, 34] and examples of PtDAs. The content and design of the PtDA were then defined based on the main categories which emerged from our focus groups and further supplemented by the results of the literature review. These elements were discussed by the steering group. The draft specifications were then defined and sent to a graphic designer who produced a draft version of the prototype.

### Alpha-testing

The draft version of the prototype was presented to patients in two focus groups (composed of respectively three and four participants), supplemented by two individual patient’s interviews. Three individual interviews with general practitioners independent of the study were also conducted. We used the same interview guide for individual interviews and focus groups. The interview guide explored content, design and practical use, allowing every participant to question each element on the tool. Written consent was obtained from the participants. The interviews were recorded, transcribed, and analysed according to the method described above.

### Final version of the prototype

The steering group met a second time to discuss and validate the adjustments suggested during the alpha-testing phase. This information was transmitted to the graphic designer.

The graphic designer produced the final version of the prototype from two versions that were identical in content but different in appearance. These two versions were submitted to the members of the steering group who validated the final version.

## Results

### Initial design

#### Literature review

We found 408 references with the search strategy. We selected 34 references. The literature review identified five randomized trials comparing antibiotic versus placebo in uUTI [5, 35–38]. These five trials were all included in a meta-analysis published in 2009 [6].

We selected four randomized trials comparing the use of an antibiotic to a non-steroidal anti-inflammatory drug (NSAID) in uUTI [9, 10, 39, 40] and four monocentric studies describing the natural history of uUTI [14, 41–43].

We selected four examples of available PtDAs: three PtDAs regarding anti-infectious treatment decisions in outpatient settings [29, 30, 44] and a fourth blank PtDA, which could be used for any shared decision-making situation.

##### Symptom duration

Untreated uUTI healed spontaneously in 50–70% of cases. Symptoms could last up to several weeks [42]. Mild to severe symptoms improved after 4.94 days in women not taking antibiotics [43]. Symptoms in women not taking an antibiotic lasted 50–60% longer than in women treated with an antibiotic to which the bacterium was susceptible [43]. Clinical resolution of symptoms was more likely in patients treated with antibiotics, with an odds ratio of 4.67 [2.34–9.35] [6].

After three days, the proportion of complete resolution of symptoms varied across studies, from 37% in patients treated with nitrofurantoin versus 20% in patients treated with placebo [5], 44% in women treated with fosfomycin versus 24% in women treated with ibuprofen [13], and 80% in women treated with norfloxacin versus 54% in women treated with diclofenac [40].

##### Risk of complications

The risk of pyelonephritis was not significantly different between patients taking an antibiotic and those taking a placebo (OR 0.33; CI [0.04–2.70]). The incidence of pyelonephritis ranged from 0 to 2.6% [6]. There was no reported case of sepsis. The French guidelines describe the risk of pyelonephritis as very low [13].

Three out of the four trials comparing antibiotic use with an NSAID in uUTI reported more pyelonephritis in women taking a NSAID compared to women taking an antibiotic [9] [10] [40].

##### Adverse reactions

The occurrence of adverse events was significantly higher in antibiotic-treated patients compared to placebo-treated patients [6].

In the case of pivmecillinam, 5–8% of adverse events were reported [38]. In a multinational trial conducted in primary care and hospital settings, patients taking single dose fosfomycin had a 6% rate of adverse events versus 8% in patients taking nitrofurantoin. The most common adverse events were gastrointestinal (nausea, vomiting, diarrhea, abdominal pain), asthenia, headache, dizziness, and vaginal discharge [5] [45]. These studies did not report any serious allergic reactions related to antibiotics.

##### Recurrence

The data did not allow for a meta-analysis on the occurrence of clinical recurrence [6]. In the study comparing nitrofurantoin to placebo the clinical recurrence rate at two weeks was between 17.6% in the placebo group and 20% in the treatment group [5]. In the study comparing pivmecillinam to placebo the recurrence rate at one month was 12–13% in both group [38]. We did not find study reporting the incidence of recurrence over longer periods.

##### Resistance

The emergence of resistance in the randomized studies varied from 0 to 45.5% in women taking an antibiotic versus 0–20% of women taking a placebo, with no significant difference [6]. In a Swedish study, antibiotic treatment for a uUTI in primary care was associated with a higher rate of bacterial resistance [19].

##### Alternative treatments

Patients taking herbal medicine did not have a different symptom course than those taking a placebo [46, 47]. There is no evidence of cranberry (*Vaccinium macrocarpon*) or hydration as an effective treatment for uncomplicated cystitis [48], 49].

#### Focus groups

Participants spoke of their personal or reported experiences with cystitis and their impact on their social and sexual lives: *“It restricts social life, because you always have to be near a bathroom (laughter)”* (P3.2), *“You don’t dare to have sex anymore”* (P4.4).

This experience touched on intimacy and could be perceived as taboo: *“It’s a feeling of guilt, actually. Well, in a way it is, because we feel that our intimacy as women is being attacked”* (P3.4).

They feared that cystitis could be complicated by renal, gynaecological, or fertility problems: *“If there’s blood in my urine it means that the kidneys must be affected” (P3.2), “It’s going to make an infection maybe a bit generalized in that area, maybe causing problems to have children...”* (P1.2).

Their knowledge about the risk factors and treatment of cystitis was part of a lay knowledge shared amongst women: *“I told my mother about it and she said: don’t worry, drink lots of water, it will pass, this antibiotic works well; because she often had it”* (P2.2).

Some participants described a feeling of infantilization and guilt during the consultation with the physician: *“The doctor or the ones I saw, made me feel like it was my fault because I didn’t wash (myself) well. Afterwards, we are told once we hold it back! We don’t have to be told every time”* (P3.2).

They wanted a personalized exchange, where they could express their experiences: *“What is important with cystitis, I learned from the doctor who took the time to explain it to me. [...] We are not in a normal state when we are sick. So he really needs to listen to us”* (P2.5).

The participants wished to clearly define cystitis and its risk factors, with a vocabulary accessible to all, without medical jargon: *“And in rather simple terms, so that everyone can understand it... Not in doctor’s language”* (P1.3). They suggested a pictorial presentation: *“The more graphic, the more people are affected”* (P1.3), accompanied by the doctor: *“The diagram is nice, but if the doctor doesn’t explain it to you, [...] she won’t understand anything”* (P3.3).

Their expectations of treatment could be the rapid relief of symptoms, or the prevention of recurrence in the longer term: *“Isn’t there something more effective and long-lasting, [...] rather than just immediately stopping the pain?”* (P1.2).

Some patients have expressed an interest in being involved in the decision related to the antibiotic: *“Do you have something to offer me that is not antibiotics? I have time now, I can stay at home, if it’s really not going well we’ll switch to antibiotics, but why don’t we test something else? Maybe there should be a second option”* (P4.4).

The action of the antibiotic was seen as magical, but could lead to side effects and resistance: *“This antibiotic was really a miracle”* (P6.1), *“Every antibiotic [...] that we swallow, we know that there are side-effects”* (P1.1).

The participants considered alternative treatments, described as natural, such as cranberry or hydration: *“Having the choice between a chemical molecule and something a bit more natural, something less harsh, I’ll take what is less harsh”* (P2.3).

### Draft version of the prototype

**Fig. 2:**
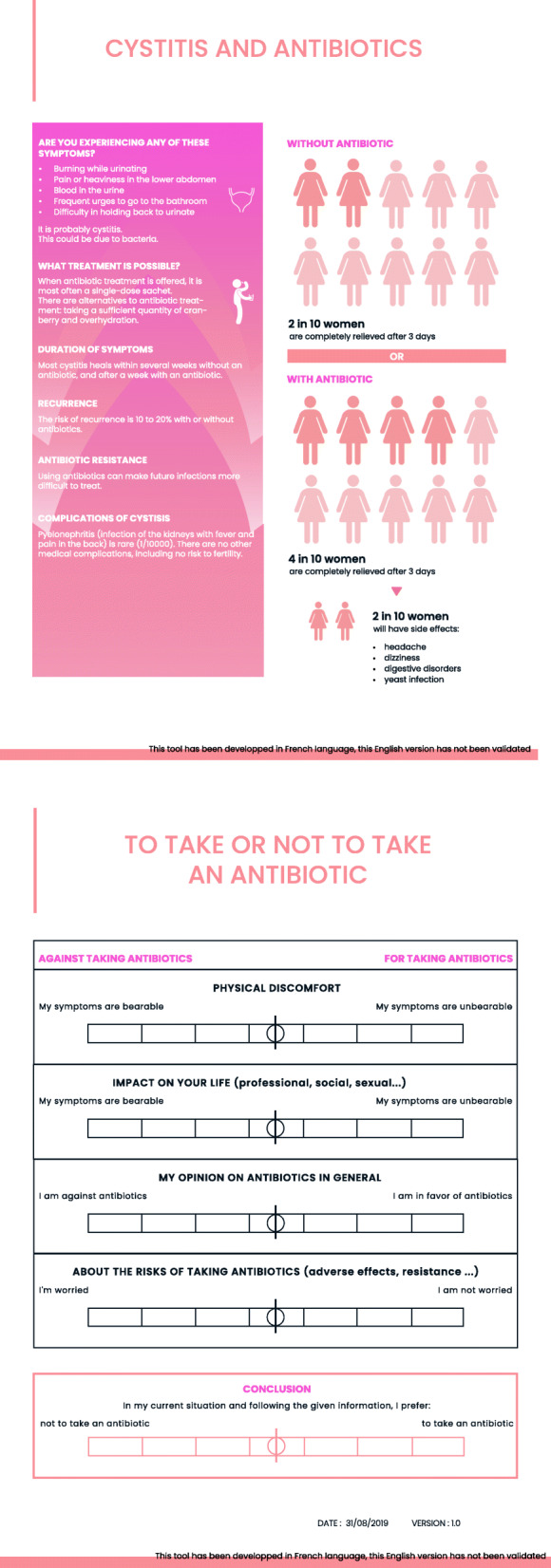
Draft version of the prototype

The draft version of the prototype (Fig. 2) included the following elements.

The title explicitly described the decision of whether to take an antibiotic or not. The elements of the PtDA were then arranged according to these two choices.

The common symptoms and etiology of uUTI were briefly described and illustrated with a diagram of the bladder.

The treatment options that were presented included antibiotic treatment, hydration, and cranberry. The practical modality of a single-dose antibiotic therapy was specified.

The average duration of symptoms, the risks of recurrence, resistance and complications were detailed according to whether the antibiotic was taken or not.

Coloured pictograms numerically represented the evolution of symptoms after three days according to the choice of treatment and the incidence of adverse effects.

The patient values to report were physical discomfort, the impact on their daily life (professional, social, sexual), their general opinion on antibiotics and their adverse effects. A free space allowed for the collection of additional value.

Deliberation was facilitated by sliders polarized according to the two options, for each expressed value. A final slider helped in the decision-making process.

The chosen format of the PtDA was a double-sided A4 sheet of paper. It was intended to be used during a discussion with the physician during the consultation and not by the patient alone.

### Alpha-testing and final version of prototype

**Fig. 3:**
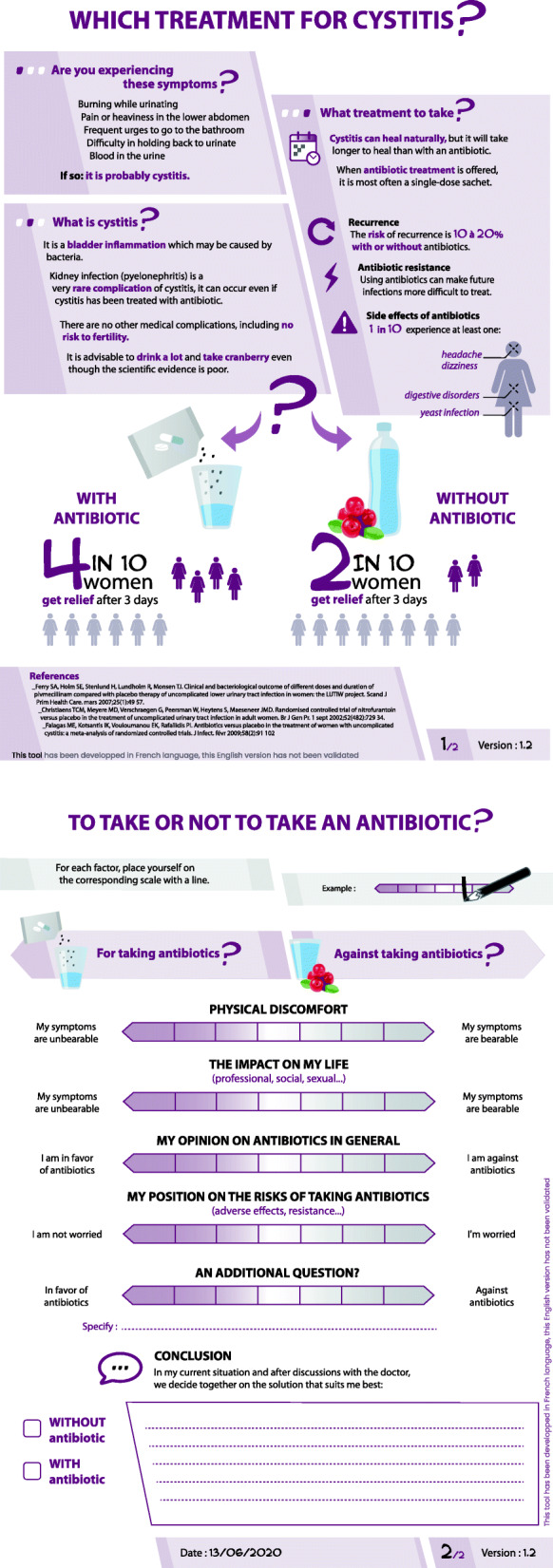
Final version of the prototype

The results of the alpha-testing phase and the second meeting of the steering group are presented in Table 1, according to the main points of the SUNDAE checklist [32]. The final prototype of the PtDA is shown in Fig. 3.

**Table 1:** Results of the alpha-testing phase and 2nd steering group

SUNDAE Check-list	*Alpha-testing Results*		Steering group
	Participants (FG + II)	Physicians (II)	Changes made
Explicit description of the decision	✗ Reformulate the title in interrogative form ✓ No need to specify the revocable nature of the decision and the possibility of re-consultation, which must be clarified orally by the doctor		⇨ Modified title ⇨ Polarized distribution of information according to the decision
Description of the health problem	✗ Need for a clearer definition of uUTI ✗ Diagram of bladder not very useful and difficult to identify	✓ Validation of symptoms description	⇨ Improved definition of uUTI, addition of the term inflammation ⇨ Removed the bladder diagram
Information on options, their benefits, risks, and consequences	✓ Overall positive to help in decision-making ✓ Layout validation ✗ Improving the visibility of adverse events and their link to antibiotics ✗ Term “several weeks” not precise enough	✓ Suitable information ✓ Information on the risk of pyelonephritis is relevant because it is not well known ✓ Interest of the precision on the absence of risk on fertility ✗ Provide information on alternative treatments to antibiotics ✗ Improve the reading of information by changing the formatting of the text	⇨ Adjusting for recurrence, complication, and adverse event rates using data from the literature ⇨ Improved description of adverse reactions ⇨ Clarification on the low level of evidence for alternative treatments (cranberry, hydration) ⇨ Improved, more spacious page layout
Numerical probabilities	✓ Validation of the pictograms ✓ Good understanding of adverse reaction data	✓ Validation of the pictograms	⇨ Adjustment using data from the literature ⇨ Addition of bibliographical references ⇨ Adding the PtDA update date
Clarification of values (implicit and explicit)	✓ Validation of the values explored ✓ Validation of the concept of slider left blank but needs to be explained		⇨ Legend for the blank slider
Guidance in deliberation	✗ Add a color gradient to the sliders, and don’t put the slider in the center by default ✓ Slider format and polarization validation ✗ Non-contributing final slider ✗ Make it clear that the patient’s decision is made orally with her doctor	✗ Coloring the sliders	⇨ Changing the slider graphics ⇨ Final slider replaced by a sentence encouraging deliberation with the doctor
Guidance in communication	✗ Reading of the PtDA to be accompanied by the doctor	✗ Fear of a difficulty of use due to lack of time, in particular with the sliders	⇨ Elements to be included in training to use the PtDA
Reading and comprehension level	✓ Understandable slider terms ✗ Prefer the term “drinking water” to “hydration”		⇨ Clarification of the definition of resistance ⇨ Replacing the term hydration
Other	✗ Enhance contrast, favor a uniform background ✓ Pink color validation	✗ Enhance contrast	⇨ Improved contrast

✗ elements to improve ✓ elements validated *FG* focus group, *II* Individual interview.

## Discussion

The literature review showed a need to increase patient’s involvement in the decision-making process for uUTI treatment. Patient’s feedbacks during focus groups confirmed this need [8] [7]. The literature review could not reveal which decision was intrinsically better than the other and confirmed the relevance of the equipoise. In 2009, a study including 1900 French general practitioners, concluded that there was a need for tools to reduce the gaps between guidelines and patient preferences [50]. Our goal is to meet this demand by providing a validated PtDA.

Hence, we developed a prototype of PtDA to help physicians and patients to make a shared decision regarding antibiotic treatment for uUTI. This prototype aims to provide support during consultations, in addition to the information delivered orally by the physician (diagnosis, risk factors, monitoring, advice for further consultation, etc.).

Of note, we recruited more patients face-to-face during medical consultations than through patient support and community groups. The ease of face-to-face recruitment was previously described in the literature [51]. Moreover, in 2016 and 2017, a law led to the creation of a new national-scale patient support and community group: France Assos Santé. This major change in the community groups field led to the need for their reorganization. This may have hindered their participation despite a strong willingness to be involved in the project.

The medium-sized focus groups in our study allowed rich and varied exchanges, including those involving intimate topics, as already described in the literature, where a focus group of four to six participants has previously been documented to facilitates communication [51]. The age and socio-professional category criteria and place of residence varied, which contributed to the expression of diverse points of view.

Some elements collected from the focus groups corresponded with the existing literature, such as representations of uUTI and its risk factors [8], representations and opinions on antibiotics [8] [7], and the impact on social or professional life [3]. Patients mentioned numerous representations relating to the gynaecological sphere, intimacy, sexuality, and fertility. Such representations are rarely found in articles regarding uUTI. Their evocation was facilitated during focus groups composed solely of women, including the observer and the investigator. The importance of representations around femininity was integrated into the PtDA using a pink/purple colour. Some members of the focus group expressed the desire to have a more gender-neutral representation. The pink colour was widely validated by the patients during the alpha-testing phase and was consequently retained.

Some of these representations correspond to known risk factors (i.e. sexual intercourse), however, the steering group decided not to mention risk factors because they were not directly involved in the decision-making process. Other representations were beliefs (risk of infertility) that were absent in the literature. Some patients wanted to be able to discuss them. This information could be added in a leaflet handed over to the patient.

Most of the patients’ values expressed in the focus groups could be integrated into the PtDA, particularly in the slider. An empty slider allows the patient to express additional values, like her expectations regarding treatment (reduction of recurrence, rapid symptom relief, etc.).

One concern expressed among the interviewed patients and doctors was the risk of pyelonephritis. There is little data on the natural course of uUTI without antibiotics, and the meta-analysis comparing antibiotics to placebo did not show a significant increase in the risk of pyelonephritis between the two [6]. However, patients treated with an anti-inflammatory drug had a higher risk of pyelonephritis compared to those treated with an antibiotic [9] [10] [40]. This increased risk could be explained by the harmful role that anti-inflammatory drugs can play in infectious diseases [40].

Some of the interviewed physicians were concerned that the use of the PtDA would lengthen the consultation. There is little evidence on the impact of shared-decision making on the length of consultations [52], but a study comparing a standard approach to the use of PtDAs in the management of depression in primary care did not show a significant difference in the length of the visit [53].

A noticeable limitation of this PtDA is the lack of medical perspectives exterior to the study. The number of interviews with physicians during the alpha-testing phase was low. As already reported in a Cochrane review, during PtDAs development, patients’ views were more often collected than those of physicians [24].

The steering group chose to present the probability of symptoms after three days. This time frame made possible to present data in the PtDAs regarding the first-line antibiotic in France (fosfomycin) and placebo. This short delay is in line with the French guidelines which recommend another consultation after three days in case of treatment failure, but also with the British guidelines proposing a 48-h delay for the delayed prescription of antibiotics. Indeed, delayed prescribing could reduce antibiotic use [54]. This option is close to immediate non-prescription and re-evaluation in case of persistent symptoms. Delaying the prescription can therefore fit in the use of the PtDA, which is not currently recommended in France [13], contrary to the United Kingdom and the Netherlands [20] [21]. Hence, our PtDA proposes not to prescribe antibiotics immediately. PtDAs have a better clinical impact when they are developed simultaneously to national guidelines [25], as NICE has been able to do [27]. Our PtDA is similar to NICE’s PtDA with regards to the information on the options, their benefits, risks, and consequences, and is based on the same literature references. On the other hand, NICE’s PtDA does not include a diagram to facilitate an appropriate understanding of numerical probabilities; besides, it does not allow patients to clarify their values nor foster deliberation, as recommended by the IPDAS [22].

Our PtDA was created in line with international standards and will soon be beta-tested in a larger study [28]. Helped by users’ feedbacks, the beta-test phase will allow to improve our tool and the global acceptance of the concept from both patient and physician perspectives.

The possibility to implement this approach in France will also be studied during the beta-test. Indeed, there may be some difficulties to implement the PtDA in clinical practice. The PtDA leads to a discussion with the patient about the choice of using antiobiotic or not, whereas actual French guidelines advise a systematic antibiotic prescription. Furthermore, systematic antibiotic prescription requires less investment from the practitioner. Nevertheless, practitioner investment can lead to better outcomes: if the practitioner is optimistic about diagnosis and prognosis, symptoms could resolve faster [43]. Thus, if our PtDA shows good implementation during the beta-test, it would lead to a large-scale study with a validated PtDA to evaluate its impact on antibiotic prescription and patients’ satisfaction. This could influence new national guidelines on cystitis for general practitioners and then facilitate the PtDAs implementation.

## Conclusion

uUTI is no longer considered as a pathology for which the symptomatology is experienced similarly for all patients and for which a single treatment should be offered. The literature review suggests that uUTI is an equipoise situation, hence, shared-decision making seems to be the best way to broach this subject. Developing a tool to help for shared-decision making in primary care consultation is necessary to move forward and to assess its efficiency compared to a systematic antibiotic treatment approach. To do so, we developed one of the first PtDA for uUTI treatment in primary care, in line with international standards.

The impact of our PtDA on patients’ satisfaction and antibiotic prescription remains to be evaluated, but its conception has already brought a lot of information on patients’ and practitioners’ perceptions of uUTI.

## Data Availability

The datasets analysed during the current study are available from the corresponding author on reasonable request.

## Appendix

*(“urinary tract infections”[MAJR] OR (“urinary tract”[TW] AND infection*[TW]) OR uti [TW] OR cystitis [TW])*

AND

*(“Anti-Bacterial Agents”[Mesh] OR antibiotic*[TW] OR antibacterial*[TW] OR anti-bacterial*[TW] OR antiinfective*[TW] OR anti-Infective*[TW])*

AND

*(“primary health care”[Mesh] OR primary health care [TW] OR “healthcare, primary”[TW] OR “care, primary”[TW] OR “primary care”[TW] OR “primary healthcare”[TW] OR “primary care setting”[TW] OR “general practitioner”[Mesh] OR “general practice”[Mesh] OR general practice*[TW].*

*OR*

*(“Meta-Analysis” [Publication Type] OR Meta-Analysis [TW] OR “Meta Analysis”[TW] OR “systematic review”[TW])*

## Abbreviations

IPDAS: International Patient Decision Aids Standards
NICE: National Institute for Health and Care Excellence
NSAID: Non-steroidal anti-inflammatory drug
PtDAs: Patient decision aids
uUTI: Uncomplicated urinary tract infection
SUNDAE: Standards for UNiversal reporting of patient Decision Aid Evaluation
